# Predictors of response in patients with active systemic JIA (SJIA) receiving canakinumab: an exploratory analysis of pooled 12-week data

**DOI:** 10.1186/1546-0096-12-S1-O12

**Published:** 2014-09-17

**Authors:** N Ruperto, HI Brunner, I Kone-Paut, B Magnusson, S Ozen, F Sztajnbok, J Anton, J Barash, F Corona, K Lheritier, C Gaillez, A Martini, D Lovell

**Affiliations:** 1 PRINTO-Istituto Gaslini, Genova, Italy; 2 PRCSG, Cincinnati, USA; 3 Novartis Pharma AG, Basel, Switzerland

## Introduction

Canakinumab (CAN), a selective, human, anti- interleukin-1β monoclonal antibody, has been shown to be efficacious in the treatment of SJIA (Ruperto et al. N Engl J Med 2012).

## Objectives

To explore baseline demographics and clinical characteristics that are most predictive of response to CAN in CAN-naïve SJIA patients during the initial 12 weeks of therapy.

## Methods

Data from 3 trials were pooled for this analysis. CAN-naïve patients (pts; n=178) aged 2–19 years with active SJIA were enrolled and received sc CAN 4 mg/kg/month; Predictors of response (according to aACR* 30, 70, and Inactive Disease [ID]) at Days (D) 15, 29, 57 and 85 were explored using univariate and multivariate logistic regression analyses. The candidate predictors (categorical variables) of CAN-response considered were: Age group, Gender, Prior NSAIDS (no/yes), Prior MTX(no/yes), Steroids (0, >0 – ≤0.4;> 0.4), Number of Active Joints(≤10, 11-≤20, >20) and Joints with Limitation of Motion (≤10, 11-≤20,>20), CRP (elevated/normal) at baseline and at D15. All candidate predictors with p<0.1 in univariate analyses were included in the multivariate analysis. *ACR response plus absence of fever.

## Results

By week 2 there was substantial clinical benefit with 102 pts (57%) and 36 pts (20%) achieving aACR70 and ID, respectively; by week 12, 108 pts (61%) had aACR70 and 50 pts (28%) ID. The multivariate analysis indicated that normal CRP at D15 is the only predictor significant (all p < 0.05) for ID at all time-points (Table 1).

**Table:** Inactive Disease - Multivariate logistic regression analysis on 12-week data

Variable*, Odds Ratio (95% CI)	Day 15	Day 29	Day 57	Day 85
**CRP at Day 15 (elevated vs normal)**	**0.20 (0.07, 0.55)**	**0.14 (0.04, 0.41)**	**0.26 (0.10, 0.66)**	**0.31 (0.12, 0.82)**

**Number of active joints (11-≤20 vs. ≤10)**	0.22 (0.03, 1.66)	0.55 (0.09, 3.41)	**0.17 (0.031, 0.97)**	0.37 (0.06, 2.10)

**Number of active joints (≤10 vs. >20)**	2.56 (0.12, 55.39)	1.53 (0.06, 37.44)	**16.10 (1.00, 258.12)**	**25.41 (1.60, 404.61)**

**Prior NSAID treatment (no vs. yes)**	2.01 (0.71, 5.71)	**9.33 (2.44, 35.68)**	**3.10 (1.03, 9.31)**	**5.31 (1.66, 17.05)**

**Steroid Level (0 vs. >0.4 mg/kg/day)**	5.48 (0.97, 31.01)	**8.89 (1.26, 62.64)**	2.98 (0.51, 17.46)	**11.16 (1.72, 72.34)**

**Steroid Level (>0.4 vs. >0-≤0.4 mg/kg/day)**	0.32 (0.08, 1.29)	0.41 (0.09, 1.82)	0.81 (0.25, 2.60)	**0.13 (0.03, 0.57)**

**Prior MTX treatment (no vs. yes)**	1.94 (0.75, 5.00)	2.78 (0.93, 8.33)	**2.79 (1.04, 7.51)**	1.77 (0.65, 4.83)

**Prior anti-TNFs treatment (no vs. yes)**	1.83 (0.52, 6.49)	3.62 (0.77, 17.00)	2.01 (0.63, 6.38)	**3.64 (1.04, 12.77)**

Values in bold are significant; *Significant in at least one time point

## Conclusion

This exploratory analysis suggests that CAN-naïve patients with normal CRP (i.e. ≤10 mg/l) at Day 15, lower baseline steroid doses, low number of active joints, no prior anti-TNF or prior NSAID use are those most likely to achieve inactive disease up to 12 weeks.

## Disclosure of interest

N. Ruperto Grant / Research Support from: To Gaslini Hospital: Abbott, Astrazeneca, BMS, Centocor Research & Development, Eli Lilly and Company, "Francesco Angelini", Glaxo Smith & Kline, Italfarmaco, Novartis, Pfizer Inc., Roche, Sanofi Aventis, Schwarz Biosciences GmbH, Xoma, Wyeth Pharmaceuticals Inc., Speaker Bureau of: Astrazeneca, Bristol Myers and Squibb, Janssen Biologics B.V., Roche, Wyeth, Pfizer, H. Brunner Consultant for: Novartis, Genentech, Pfizer, UCB, AstraZeneca, Biogen, Boehringer-Ingelheim, Regeneron, Paid Instructor for: Novartis, Speaker Bureau of: Novartis, Genentech, I. Kone-Paut Grant / Research Support from: SOBI, Chugai, Consultant for: Pfizer, SOBI, Novartis, AbbVie, Cellgene, Chugai, B. Magnusson: None declared., S. Ozen Consultant for: Novartis (Turkey), Speaker Bureau of: Speaker’s fee from SOBI, F. Sztajnbok Grant / Research Support from: Institutional grant (UERJ) for participating in the canakinumab trial., Speaker Bureau of: Novartis-Brasil, J. Anton Consultant for: Novartis, Speaker Bureau of: Novartis, J. Barash Grant / Research Support from: Investigator in the Canakinumab study sponsored by Novartis, F. Corona: None declared., K. Lheritier Shareholder of: Novartis, Employee of: Novartis Pharma AG, C. Gaillez Employee of: Novartis Pharma AG, A. Martini Grant / Research Support from: Bristol Myers and Squibb, Centocor Research & Development,Glaxo Smith & Kline, Novartis, Pfizer Inc, Roche, Sanofi Aventis, Schwarz Biosciences GmbH, I declare that the Gaslini Hospital which is the public Hospital where I work as full time employee has received contributions to support the PRINTO research activities from the industries above mentioned. OLD: Francesco Angelini S.P.A., Janssen Biotech Inc, Abbott. , Consultant for: Bristol Myers and Squibb, Centocor Research & Development, Glaxo Smith & Kline, Novartis, Pfizer Inc, Roche, Sanofi Aventis, Schwarz Biosciences GmbH, I declare that the Gaslini Hospital which is the public Hospital where I work as full time employee has received contributions to support the PRINTO research activities from the industries above mentioned. , Speaker Bureau of: Abbott, Bristol Myers Squibb, Astellas, Boehringer, Italfarmaco, MedImmune, Novartis, NovoNordisk, Pfizer, Sanofi, Roche, Servier, D. Lovell Grant / Research Support from: National Institutes of Health- NIAMS , Consultant for: Astra-Zeneca, Centocor, Amgen, Bristol Meyers Squibb, Abbott, Pfizer, Regeneron, Roche, Novartis, UBC, Forest Research Institute, Horizon, Johnson & Johnson, Speaker Bureau of: Novartis, Roche.

